# Normalized GNSS Interference Pattern Technique for Altimetry

**DOI:** 10.3390/s140610234

**Published:** 2014-06-11

**Authors:** Miguel Angel Ribot, Jean-Christophe Kucwaj, Cyril Botteron, Serge Reboul, Georges Stienne, Jérôme Leclère, Jean-Bernard Choquel, Pierre-André Farine, Mohammed Benjelloun

**Affiliations:** 1 Electronics and Signal Processing Laboratory (ESPLAB), École Polytechnique Fédérale de Lausanne (EPFL), Maladière 71B (Microcity), CH-2002 Neuchâtel, Switzerland; E-Mails: cyril.botteron@epfl.ch (C.B.); jerome.leclere@epfl.ch (J.L.); pierre-andre.farine@epfl.ch (P.-A.F.); 2 Laboratoire d'Informatique, Signal et Image de la Côte d'Opale (LISIC), Univ Lille Nord de France, F-59000 Lille, France. Université du Littoral Côte d'Opale (ULCO), 50, rue Ferdinand Buisson, BP719-62228 Calais cedex, France; E-Mails: kucwaj@lisic.univ-littoral.fr (J.-C.K.); serge.reboul@univ-littoral.fr (S.R.); stienne@lisic.univ-littoral.fr (G.S.); jean-bernard.choquel@univ-littoral.fr (J.-B.C.); mohammed.benjelloun@lisic.univ-littoral.fr (M.B.)

**Keywords:** GNSS signal processing, reflectometry, GNSS-R, interference pattern technique

## Abstract

It is well known that reflected signals from Global Navigation Satellite Systems (GNSS) can be used for altimetry applications, such as monitoring of water levels and determining snow height. Due to the interference of these reflected signals and the motion of satellites in space, the signal-to-noise ratio (SNR) measured at the receiver slowly oscillates. The oscillation rate is proportional to the change in the propagation path difference between the direct and reflected signals, which depends on the satellite elevation angle. Assuming a known receiver position, it is possible to compute the distance between the antenna and the surface of reflection from the measured oscillation rate. This technique is usually known as the interference pattern technique (IPT). In this paper, we propose to normalize the measurements in order to derive an alternative model of the SNR variations. From this model, we define a maximum likelihood estimate of the antenna height that reduces the estimation time to a fraction of one period of the SNR variation. We also derive the Cramér–Rao lower bound for the IPT and use it to assess the sensitivity of different parameters to the estimation of the antenna height. Finally, we propose an experimental framework, and we use it to assess our approach with real GPS L1 C/A signals.

## 1. Introduction

Global Navigation Satellite Systems reflectometry (GNSS-R) is a well-established method for remotely sensing many relevant geophysical properties of the reflection surfaces. GNSS-R was first proposed within the frame of the PAssive Reflectometry Interferometric System (PARIS) project as a bistatic radar remote sensing technique for ocean altimetry using the L-band GPS signal [1]. Since then, GNSS-R has been demonstrated to be useful in other applications, such as monitoring water levels and snow height with a ground approach [2–4]. In this approach, the antenna, situated on a mast, receives a direct GNSS signal coming from the satellite and a nadir signal reflected by the observed surface. Assuming that the antenna position is known, we can compute the position of the surface of reflection. This approach provides precise localization and dating of the measures that allows for spatio-temporal comparison of water levels and snow cover, respectively [5–8]. These parameters are very important for flood monitoring and avalanche prevention, as well as for hydroelectric companies. Furthermore, the approach is noninvasive and can be easily implemented on a portable instrument and embedded in a vehicle with a mast [9].

GNSS-R altimetry can be carried out in two different ways, depending on the ranging principle: code altimetry and phase altimetry [10]. With code altimetry, only the GNSS code delay difference between the direct and reflected signals is used. With phase altimetry, the phase of the signal is also used for computing this delay difference [9]. The interference pattern technique (IPT) considers the behavior of the signal-to-noise ratio (SNR) of the received GNSS signal as a function of the satellite elevation [2,11]. The direct and reflected GNSS signals are combined at the antenna. Due to their different phase variations, the SNR oscillates at a rate proportional to the distance between the antenna and the surface of specular reflection. Unlike satellite or airborne reflection scenarios, ground GNSS receivers observe a coherent interference pattern if we consider a flat reflecting surface (compared to the carrier wavelength) on an area corresponding to the first Fresnel zone. A few previous works can be found analyzing the accuracy of these GNSS-R altimetry techniques [12–15]. Initial works proposed simple analytical models to describe the altimetry precision as a function of system/instrument parameters [12]. These methods rely on a considerable number of assumptions that might hold only for some specific scenarios and provide a pessimistic bound on the achievable precision. In [13], the authors proposed a Cramér–Rao lower bound (CRLB) closed expression for code altimetry. The CRLB is a statistical tool that provides a lower bound on the achievable estimation error for any unbiased estimator. A new derivation was proposed in [15] to compute the CRLB for code altimetry and a specific set of measurement data under multiple SNR scenarios.

Unfortunately, one of the main drawbacks of the IPT is that very long measurement times are usually needed. The observed SNR oscillates with the variation of the satellite elevation, but satellite elevation varies slowly, thus long measurement times are required to estimate the SNR frequency of oscillation. To reduce the estimation time to a fraction of one period of the SNR variations, we propose an alternative model for the measured SNR. This normalized model is based on the normalization of the measured signal amplitudes and is possible only after an initial calibration step. This calibration step consists of varying the antenna height of the receiver a value *dh* in order to obtain the minimum and maximum value of SNR for a given satellite elevation. Using the normalized model, we define a maximum likelihood estimate of the antenna height that allows for the reduction of the required estimation time to a fraction of one period of the SNR variation. We also derive the minimum antenna variation range *dh* as a function of the satellite elevation and deduce from this function the minimum observation time as a function of the satellite elevation rate. In addition, we derive the CRLB for the IPT and use it to assess the sensitivity of different parameters to the estimation of the antenna height. Finally, we propose a novel experimental framework, which we use to assess our approach with real signals.

This paper is organized as follows: Section 2 describes the interference pattern problem. The considered signal model is introduced as a function of the receiver height. The proposed estimator is presented in Section 3. Section 4 includes the derivation of the CRLB for the IPT and the proposed estimator performance assessment using synthetic signals. In Section 5, the proposed experimental framework is described, and the results obtained with real GPS L1 C/A signals within this framework are presented. Finally, Section 6 summarizes the paper, highlighting its main conclusions.

## 2. Interference Pattern Problem

**Figure 1.**
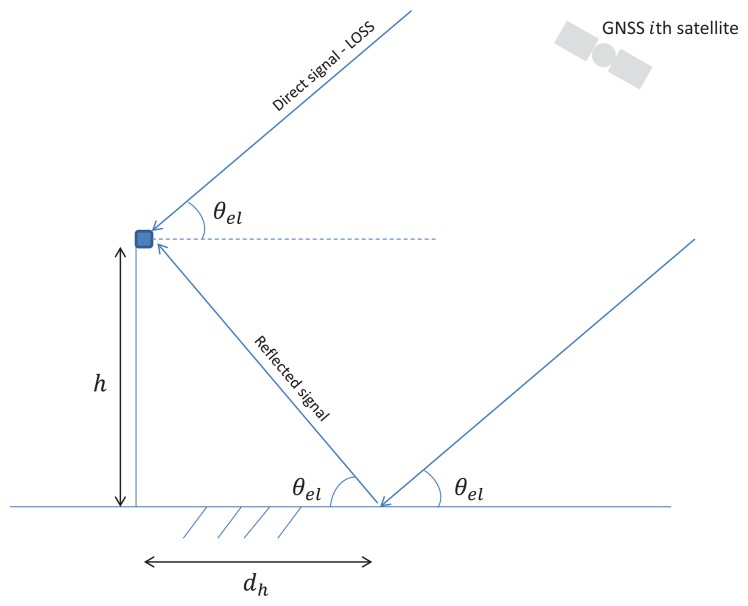
Reflectometry principle for an antenna on a mast.

We show in Figure 1 the reflectometry principle for an antenna situated on a mast of height *h*. In our approach, we take into account the subset of satellites *M* that have a specular reflection on the surface to analyze. The antenna receives *M* scaled, time-delayed and Doppler-shifted signals with known signal structures. Each signal corresponds to the line-of-sight or direct signal (*s_D_i__*) plus its corresponding specular reflection (*s_R_i__*). The overall received signal can be modeled as:

(1) $$\begin{array}{ll} x(t) & =\overset{M}{\underset{i=1}{\text{∑}}}\left(s_{D_{i}}+s_{R_{i}}\right)+n(t)\\ & =\overset{M}{\underset{i=1}{\text{∑}}}A_{D_{i}}c_{i}\left(t-\tau_{D_{i}}\right)\cos\left(2\pi \left(f_{RF}+f_{d_{i}}\right)t+\phi_{D_{i}}\right)\\ & +\overset{M}{\underset{i=1}{\text{∑}}}A_{R_{i}}c_{i}\left(t-\tau_{R_{i}}\right)\cos\left(2\pi \left(f_{RF}+f_{d_{i}}\right)t+\phi_{D_{i}}+\phi_{R_{i}}(t)\right)+n(t), \end{array}$$

where *f_RF_* is the carrier frequency, *f_d_i__* the Doppler frequency shift of the *i*-th satellite, *ϕ_D_i__* the receiver clock phase offset, *ϕ_R_i__*(*t*) the phase delay between the direct and reflected signals as a function of time, *τ_D_i__*, *τ_R_i__* the time-delays, *c_i_*(*t*) the pseudorandom code sequence, *A_D_i__* and *A_R_i__* the amplitudes of the direct and reflected received signals and *n*(*t*) zero-mean additive Gaussian noise with variance $\sigma_{n}^{2}$. In this paper, we will assume that the variations of *A_D_i__* and *A_R_i__* are negligible during the short periods of observation considered, e.g., a few minutes. For long observation times, *A_D_i__* and *A_R_i__* will change with time as a function of the satellite elevation due to the variation of the received power and the antenna footprint. The time dependence of *τ_D_i__*, *τ_R_i__*, *f_d_i__* and *ϕ_D_i__* has been neglected for simplicity in Equation (1), since their variation over time will be compensated for by the receiver's tracking stage with little impact on the proposed analysis.

In our current study, we will consider only the processing of the GPS L1 C/A signals. In this case, for an antenna mounted on a mast a few meters above the reflecting surface, the difference in GNSS signal path between the direct and the reflected signals will be small compared to the chip duration of the code. Thus, we can assume that *R_i_*(*t* − *τ_R_i__*) ≈ *R_i_*(*t* − *τ_D_i__*), where *R_i_*(*τ*) is the autocorrelation of *c_i_*(*t*). According to the geometry depicted in Figure 1, it is easy to show that:

(2) $$\phi_{R_{i}}(t)=\frac{4\pi }{\lambda_{L1}}h\sin\left(\theta_{el_{i}}(t)\right)$$

(3) $$d_{h_{i}}(t)=\frac{h}{\tan\left(\theta_{el_{i}}(t)\right)}$$

where *θ_el_i__*(*t*) is the elevation of the *i*-th satellite at instant *t*, *λ_L_*_1_ = 19.042 cm is the GPS L1 C/A carrier wavelength, *h* is the height of the antenna and *c* represents the speed of light. In the following, for notation simplicity, we will drop the satellite index *i*, since we will focus on the processing of the direct and reflected signals coming from a single satellite. In this case, by using some trigonometry, we can express *x*(*t*) as:

(4) $$x(t)=A_{G}(t)c\left(t-\tau \right)\cos\left(2\pi \left(\left(f_{RF}+f_{d}\right)t\right)-\phi_{G}(t)\right)$$

where:

(5) $$\phi_{G}(t)=\phi_{D}+\arctan\left(\frac{A_{R}\sin\left(\phi_{R}(t)\right)}{A_{D}+A_{R}\cos\left(\phi_{R}(t)\right)}\right)$$

(6) $$A_{G}(t)=\sqrt{A_{D}^{2}+A_{R}^{2}+2A_{D}A_{R}\cos\left(\phi_{R}(t)\right)}$$

and *A_G_*(*t*) represents the magnitude of the composite signal, while *ϕ_G_*(*t*) represents its phase. In practice, in a GNSS receiver, the SNR is estimated after the signal down-conversion and correlation with a local code replica. In this case, the SNR is proportional to $A_{G}^{2}(t)$, the squared amplitude of the received signal. In this context, we see from Equations (2) and (6) how $A_{G}^{2}(t)$ evolves as a cosine of the sine of the satellite elevation. The frequency of this cosine, $\frac{2h}{\lambda_{L1}}$, is proportional to the antenna height. This means that by estimating the frequency of the observed SNR, we can obtain the height of the receiver.

In order to get an accurate estimate of the frequency of cos(*ϕ_R_*(*t*)) with classic approaches, one must observe at least one period of the signal. For a given initial elevation *θ_el_*(*t*_0_), we define Δ*θ_el_*, the satellite elevation variation required to observe one period of the signal. Based on Equation (2) and using trigonometric identities, we can thus write:

(7) $$\frac{c}{2f_{L1}h}-2\sin\left(\frac{\Delta \theta_{el}}{2}\right)\;\cos\left(\theta_{el}(t_{0})+\frac{\Delta \theta_{el}}{2}\right)=0$$

**Figure 2.**
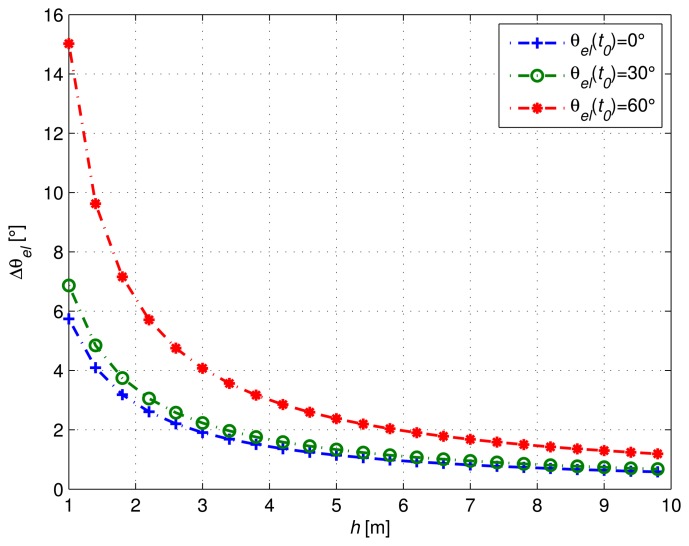
Satellite elevation variation as a function of the antenna height.

Figure 2 shows the corresponding elevation variation required according to antenna height, for different *θ_el_*(*t_0_*) values. In particular, we can see that for a height of 3 m, one period of the cosine can be observed for a satellite elevation variation of at least 2° when *θ_el_* (*t_0_*) is close to 0°. If we consider, for example, a mean satellite elevation speed *ω_el_* = 10^−3^ °/s, we must thus wait at least 33 min to observe one period of the signal. In the next section, we propose a normalization procedure to decrease the required observation period.

## 3. Proposed Approach

### 3.1. Normalization of the GNSS Signal Amplitudes

As described in Equation (2), the phase *ϕ_R_*(*t*) is a function of the satellite elevation and of the antenna height. Since the satellite elevation evolves slowly, we propose a calibration procedure that uses instead a variation of the antenna height. From Equation (6), the minimum and maximum values of *A_G_*(*t*) can be obtained when cos(*ϕ_R_*(*t*)) is equal to −1 or one, respectively. They are defined by the following expressions:

(8) $${\left(A_{G,\min}\right)}^{2}=A_{D}^{2}+A_{R}^{2}-2A_{D}A_{R}$$

(9) $${\left(A_{G,\max}\right)}^{2}=A_{D}^{2}+A_{R}^{2}+2A_{D}A_{R}$$

From these two equations, we can deduce the sum of the square of the amplitudes and their product as:

(10) $$A_{D}^{2}+A_{R}^{2}=\frac{A_{G,\max}^{2}+A_{G,\min}^{2}}{2}$$

(11) $$2A_{D}A_{R}=\frac{A_{G,\max}^{2}-A_{G,\min}^{2}}{2}$$

Therefore, upon substituting Equations (11) and (10) into Equation (6), the single unknown parameter to estimate will be the phase delay *ϕ_R_*(*t*), which is proportional to the height of the antenna and the sine of the known satellite elevation angle.

In order to always get the maximum and minimum value of *A_G_*, the variation of *ϕ_R_* should be greater than or equal to 2*π*. According to Equation (2), the minimum variation of the antenna height *dh* should thus be equal to:

(12) $$dh_{\min}=\frac{\lambda_{L1}}{2\sin\left(\theta_{el}\right)}$$

**Figure 3.**
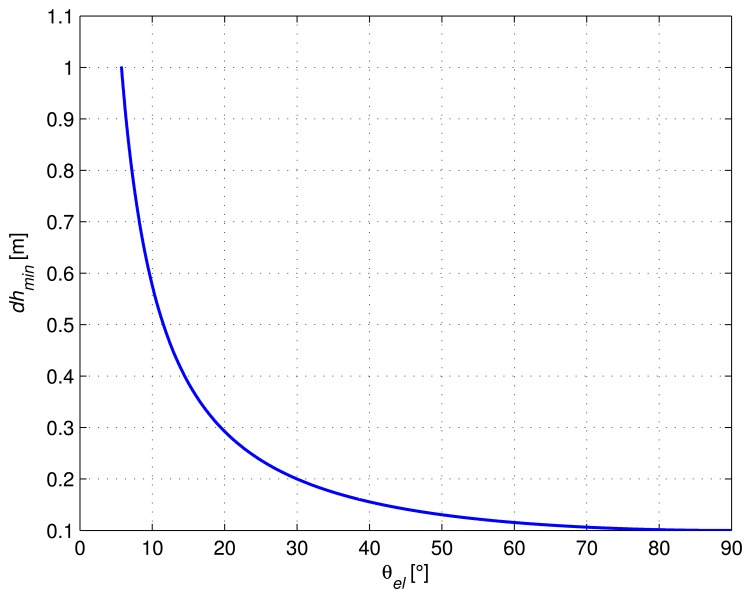
Antenna variation as a function of the satellite elevation.

In Figure 3, we show the value of *dh_min_* as a function of the satellite elevation. From this figure, we can see that a variation in the antenna height of 0.5 m is sufficient to observe a maximum and a minimum value for *A_G_* when the satellite elevation is higher than 12°.

### 3.2. Frequency Estimation

After down-conversion, the received signal is correlated with a local code replica. For the following derivation, we define the samples *y*[*n*] as the noisy post-correlation measurements obtained every *t* = *nT_int_*, where *T_int_* is the coherent integration time. From Equation (6), we can define *y*[*n*] as:

(13) $$y[n]=A_{G}[n]+w[n]$$

(14) $$=\sqrt{A_{D}^{2}+A_{R}^{2}+2A_{D}A_{R}\cos\left(\phi_{R}[n]\right)}+w[n]$$

where *w*[*n*] is the resulting zero-mean additive white Gaussian noise (AWGN) with power $\sigma_{n}^{2}$. Note that if the pre-correlation noise samples are colored (e.g., due to front-end imperfections or interfering signals), we assume that spectral whitening has been used to optimally process the RF front-end output samples (see, e.g., [16]). From Equation (2), we note that *ϕ_R_*[*n*] evolves linearly as a function of sin(*θ_el_* [*n*]), with a constant factor $\beta =4\pi \frac{f_{L1}}{c}h.$

Let us define:

(15) $$\phi_{R}^{\text{model}}[n]=\beta \sin\left(\theta_{el}[n]\right)$$

Therefore, the factor *β* defines the frequency of cos(*ϕ_R_*[*n*]), and its evolution is defined as a function of the sine of the elevation. The satellite elevation *θ_el_*[*n*] is obtained from the current GPS ephemeris data and the estimated position of the GNSS receiver. In order to estimate *β*, after calibration, we can define the following model for *A_G_*[*n*]:

(16) $${\tilde{A}}_{G}[n]=\sqrt{\frac{A_{G,\max}^{2}+A_{G,\min}^{2}}{2}+\frac{A_{G,\max}^{2}-A_{G,\min}^{2}}{2}\cos\left(\phi_{R}^{\text{model}}[n]\right)}$$

and derive the maximum likelihood estimate of *β* for *N* measurements as:

(17) $$\hat{\beta }=\underset{\beta }{\underset{︸}{\text{argmin}}}\left\{\sum_{n=1}^{N}{\left(y[n]-{\tilde{A}}_{G}[n]\right)}^{2}\right\}$$

Finally *ĥ* a function of *β̂* defined by

(18) $$\hat{h}=\frac{\hat{\beta }c}{4\pi \;f_{L1}}$$

In the next section, we will derive the CRLB for Equation (14) in order to make a feasibility study and assess the expected performance of the proposed approach.

## 4. Performance Assessment

### 4.1. Cramér-Rao Lower Bound

We are interested in assessing the maximum theoretical accuracy that can be obtained when estimating the receiver height *h*. Unfortunately, Equation (14) is highly nonlinear, which makes it difficult to directly assess the impact of its different parameters over the estimation error. This nonlinearity is due mainly to the cosine function in the expression and is exacerbated by the presence of the root mean square. Instead, we propose to compute the CRLB for the signal model under consideration. The CRLB provides a lower bound on the variance of any unbiased estimator and, thus, will allow us to assess the performance of our estimator [17].

The signal model considered for *A_G_* [*n*] is provided in Equation (14). In order to provide more insightful results, we will express the reflected signal amplitude as *A_R_* = *αA_D_*, where *α* represents the attenuation coefficient due to reflection, assumed to be real and less than or equal to one. In addition, we define $\gamma [n]≜\frac{4\pi }{\lambda }\sin\left(\theta_{el}[n]\right)$. Thus, we obtain:

(19) $$y\left[n;\text{ξ}\right]=A_{G}\left[n;\text{ξ}\right]+w[n]=A_{D}\sqrt{1+\alpha^{2}+2\alpha \cos\left(\gamma [n]h\right)}+w[n]$$

where ***ξ*** = [*A_D_*, *α*, *h*] *^T^* is our unknown deterministic parameter vector and *w*[*n*] are zero-mean AWGN samples with variance $\sigma_{n}^{2}$. The CRLB for ***ξ*** can be expressed as [17]:

(20) $$\text{var}\left({\hat{\text{ξ}}}_{i}\right)\geq {\left[{\text{I}}^{-1}\left(\text{ξ}\right)\right]}_{ii}$$

where **I** (***θ***) is the Fisher information matrix (FIM). A full account of the derivation of the FIM for the considered signal model of *y*[*n*; ***ξ***] can be found in Appendix 1. The obtained FIM is:

(21) $$\text{I}(\text{ξ})=\frac{1}{\sigma_{n}^{2}}\left[\begin{array}{ccc} \frac{1}{A_{D}^{2}}\overset{N-1}{\underset{n=0}{\text{∑}}}A_{G}^{2}\left[n;\text{ξ}\right] & A_{D}\overset{N-1}{\underset{n=0}{\text{∑}}}\left(\alpha +\cos\left(\gamma [n]h\right)\right) & -A_{D}\alpha \overset{N-1}{\underset{n=0}{\text{∑}}}\gamma [n]\sin\left(\gamma [n]h\right)\\ A_{D}\overset{N-1}{\underset{n=0}{\text{∑}}}\left(\alpha +\cos\left(\gamma [n]h\right)\right) & A_{D}^{4}\overset{N-1}{\underset{n=0}{\text{∑}}}{\left[\frac{\left(\alpha +\cos\left(\gamma [n]h\right)\right)}{A_{G}\left[n;\text{ξ}\right]}\right]}^{2} & -A_{D}^{4}\alpha \overset{N-1}{\underset{n=0}{\text{∑}}}\frac{\gamma [n]\left(\alpha +\cos\left(\gamma [n]h\right)\right)\sin\left(\gamma [n]h\right)}{A_{G}^{2}\left[n;\text{ξ}\right]}\\ -A_{D}\alpha \overset{N-1}{\underset{n=0}{\text{∑}}}\gamma [n]\sin\left(\gamma [n]h\right) & -A_{D}^{4}\alpha \overset{N-1}{\underset{n=0}{\text{∑}}}\frac{\gamma [n]\left(\alpha +\cos\left(\gamma [n]h\right)\right)\sin\left(\gamma [n]h\right)}{A_{G}^{2}\left[n;\text{ξ}\right]} & A_{D}^{4}\alpha^{2}\overset{N-1}{\underset{n=0}{\text{∑}}}{\left[\frac{\gamma [n]\sin\left(\gamma [n]h\right)}{A_{G}\left[n;\text{ξ}\right]}\right]}^{2} \end{array}\right]$$

The CRLB for *h* can be obtained by computing [**I**^−1^ (***θ***)]_33_, resulting in:

(22) $${\text{var}}_{\text{CRB}}\left(\hat{h}\right)\geq \frac{1}{{\text{SNR}}_{D}}\cdot g\left(h,\alpha ,\lambda ,\Delta {\text{θ}}_{el}\right)$$

where ${\text{SNR}}_{D}=\frac{2\sigma_{n}^{2}}{NA_{D}^{2}}$ is the post-correlator SNR when only the direct signal is received. For simplicity, the *g*() function is used to represent the multiple terms of [**I**^−1^ (***θ***)]_33_. *Δ* ***θ****_el_* = {*θ_el_* [0], *θ_el_* [1],…, *θ_el_* [*N*−1]} is the satellite elevation span covered by *N* measurements with 0 ≤ *θ_el_*[*n*] ≤ 90°.

The purpose of the following discussion is to identify the effects of {*h, α, λ, Δ****θ****_el_*} parameters through simulation. In order to highlight the effect of a specific parameter, the CRLB is computed for different values of the parameter of interest, while the rest are kept fixed. We have set *λ* = *λ_L_*_1_, corresponding to the GPS C/A L1 wavelength, a sampling period *T_s_* = 1 sample/s and $\alpha =\sqrt{0.7}$ (water surface scenario for a typical smooth sea [18]) in all of the considered cases, unless otherwise specified.

### 4.2. Assessing the Initial Elevation on the CRLB

**Figure 4.**
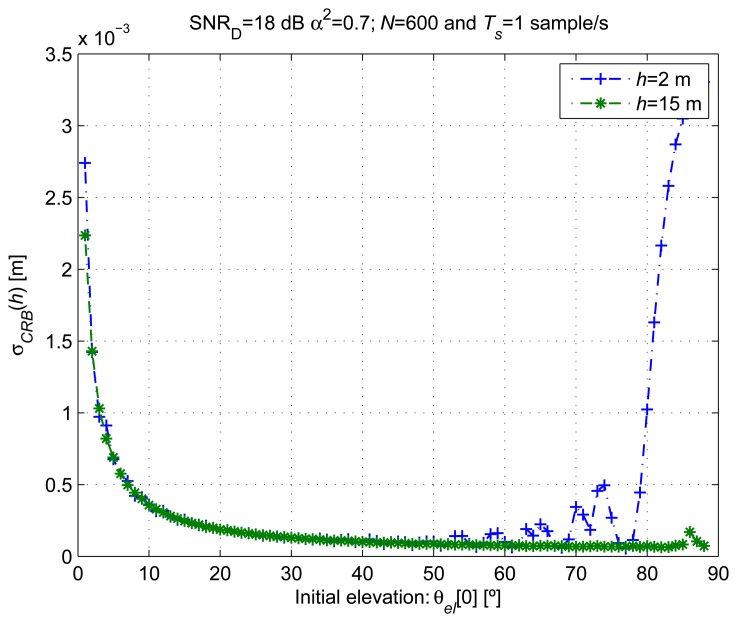
Cramér-Rao lower bound (CRLB) for different initial satellite elevation angles and receiver heights.

Figure 4 shows the $\sigma_{\text{CRB}}(h)≜\sqrt{{\text{var}}_{\text{CRB}}\left(\hat{h}\right)}$ for different satellite initial elevations (*θ_el_* [0]) and antenna heights of *h* = 2 m and *h* = 15 m. Elevation variations of 3° were covered by *N* = 600 observations starting from the different *θ_el_* [0] values. A reference SNR*_D_* = 18 dB was considered. The CRLB is computed from the probability density function (pdf) of the data observations. The estimation accuracy, lower-bounded by the CRLB, is related to the dependency of the data pdf on the unknown parameters (**ξ**). The CRLB is higher when the dependency between the observations and the parameter to estimate is weak.

**Figure 5.**
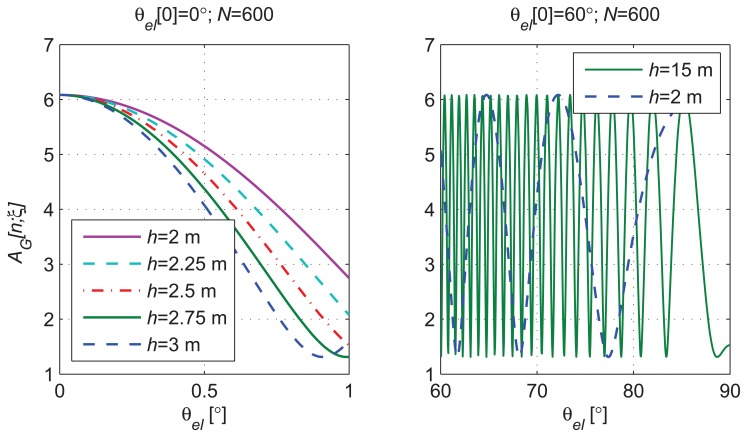
Examples of *A_G_* evolution for different receiver heights, in the absence of noise: (**Left**) *A_G_* for different elevation angle variations and receiver heights; (**Right**) *A_G_* for elevation angle variations between 60° and 90° and receiver heights of 2 m and 15 m.

Remarks:

- When *θ_el_*[0] is close to zero, the CRLB is several times higher than for higher *θ_el_*[0] values and almost independent of *h*. In order to explain this behavior, we have shown in Figure 5Left the *A_G_* evolution for different *h* values over Δ*θ_el_* = 1° for *θ_el_* [0] < 1°. The dependency between *h* and the observations becomes smaller when small *θ_el_*[0] values are considered, even for the same number of samples.
- When *A_G_* [*n*; ***ξ***] (see Equation (19)) is at its maximum or minimum value, for a small period of observation, the signal tends to a constant equal to $A_{D}\sqrt{1+\alpha^{2}+2\alpha }$ or $A_{D}\sqrt{1+\alpha^{2}-2\alpha }$, respectively. In this region, the signal evolution can be assumed to be monotone with almost null variation. The dependency between the observations and *h* decreases with the absolute value of the slope of *A_G_* [*n*; ***ξ***] during the period of observation. In this case, the CRLB is as high as the slope of the signal is low.
- We show in Figure 5Right that the frequency of the cosine evolution of *A_G_* [*n*;***θ***] decreases when *θ_el_* tends to 90°. For *h* = 2 m and an observation interval of 600 s, we observe periodic CRLB increases with the satellite elevation when Equation (19) includes a minimum or a maximum. This situation corresponds to the peaks that appear in Figure 4 for *h* = 2 m and *θ_el_*[0] > 50°. For *h* =*15 m* and an observation interval of 600 s, the minimum frequency of Equation (19) is too high to have a constant signal evolution in a period of observation. Thus, the CRLB for *h* =*15 m* does not present the peaks as when *h* = 2*m*.

From the observed behavior of the CRLB, we prefer using the proposed estimator with satellite elevations between 10° and 70° and antenna heights greater than 2 m to obtain better estimation accuracy. For low elevations, the CRLB is indeed high, independent of the antenna height. For elevations close to 90°, the CRLB increases for low antenna heights, because just a small portion of the signal *A_G_* [*n*; ***ξ***] oscillation, considerably less than a period, is observed. As expected, this effect is exacerbated when the *ω_el_* is lower, and a shorter evolution of *A_G_* [*n*; ***ξ***] is observed for the same measurement period.

### 4.3. Assessing the Elevation Rate on the CRLB

**Figure 6.**
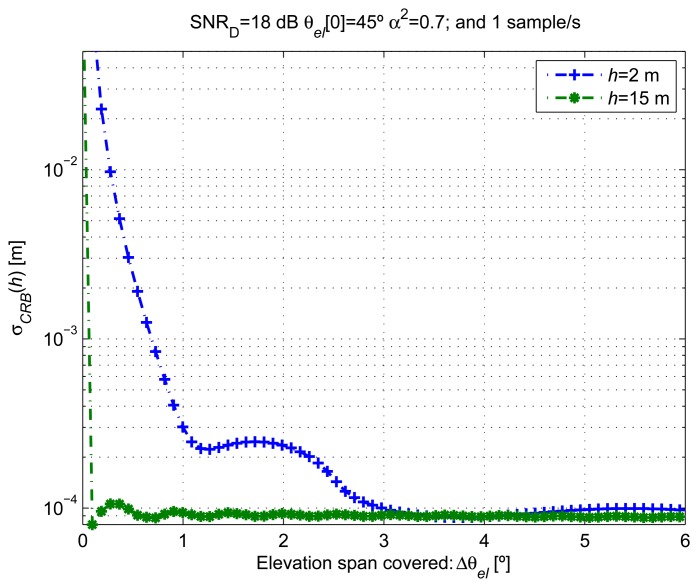
CRLB for different elevation angle variations and receiver heights.

Figure 6 shows the *σ_CRB_*(*h*) computed for different elevation variations *Δ****θ*** *_el_* from 0° to 6° with a fixed *θ_el_* [0] = 45°. For receiver heights *h* = 2 m and *h* = 15 m, *θ_el_* [0] = 45° was selected, since it approximately corresponds to a minimum of the *A_G_* [*n*; ***ξ***] evolution, which corresponds to the worst scenario for the estimation of its frequency of oscillation. These *Δ****θ****_el_* values were generated by assuming constant *ω_el_* up to 10 × 10^−3^ °/s for a fixed measurement period of 600 s and a sampling rate of one sample/s. From the figure, we observe that by increasing Δ***θ****_el_*, the variance decreases quickly at first, until an entire period of oscillation of the signal model is covered. This relation can be used to set the duration of the measurement time for our estimator, since we are interested in achieving the maximum possible accuracy with the minimum number of data observations. Unfortunately, the period of oscillation of our model depends on the true value of *h* and *ω_el_*. The measurement time required to achieve a certain target accuracy will depend on *ω_el_*, and in general, for faster *ω_el_*, shorter measurement periods will be required to achieve a similar estimation accuracy.

### 4.4. Assessing the Impact of Amplitude Ratio α on the CRLB

**Figure 7.**
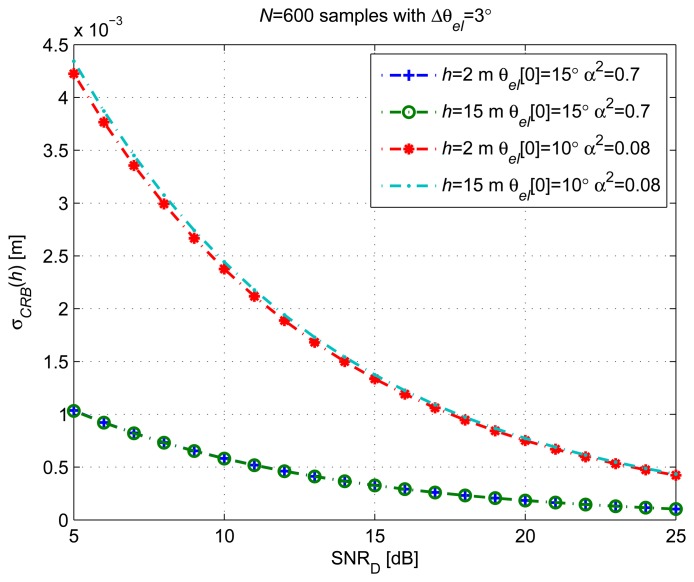
*σ_CRB_*(*h*) for different receiver heights and reflected/direct amplitude ratios (*α*) *versus* SNR.

Figure 7 shows the *α_CRB_*(*h*) obtained for different receiver heights (*h* = 2 m and *h* = 15 m) and reflected/direct amplitude ratios (*α*) *versus* SNR*_D_*. The coefficient *α* can be interpreted as the square-root magnitude of the polarization-dependent reflection coefficient. For right-hand circular polarization (RHCP) of the incident signal and left-hand circular polarization (LHCP) of the reflected signal, *α* can be calculated as a function of *θ_el_* and the dielectric constant of the scattering surface, assuming a smooth surface [18]. Two values for *α* have been selected. The first value, $\alpha w=\sqrt{0.7}$, corresponds to sea water, with a typical dielectric constant *ε_W_* = 73.0 + *i*57.5 for *λ_L_*_1_ = 0.19 m and an *θ_el_* = 15° [18]. The second value, $\alpha_{S}=\sqrt{0.08}$, corresponds to fresh snow at −2 °C, with *ε_S_* = 1.45 + *i*2.76 × 10^−4^ for a *θ_el_* ≃ 10° [19,20]. The *Δ****θ****_el_* considered covers a satellite elevation variation of 3° with an *θ_el_* [0] = 15°, *N* = 600 samples and *T_s_* = 1 sample/s.

The figure shows that the estimation error of *h* is inversely proportional to *α*. Some small differences are observed between different *h* values. These differences appear due to the differences on the oscillation period covered in each case by the interference pattern signal *A_G_*[*n*; ***ξ***] used for computing the CRLB.

### 4.5. Estimator Performance Evaluation with Synthetic Data

In order to complement the CRLB analysis, we want to assess the performance of the estimator proposed in Section 3 with synthetic data generated using real measurements of the satellites elevation (*θ_el_*). These data were generated using the signal model defined in Equation (19) with a constant SNR*_D_*=18 dB for the direct signal and a sampling period of 1 s. The SNR*_D_* value selected is typically achieved at the post-correlation stage and matches the SNR corresponding to a carrier-to-receiver noise density *C*/*N*_0_ = 45 dB-Hz with a front-end bandwidth of 3 MHz, sampling at the Nyquist frequency, and a coherent integration time of 1 ms. The power ratio between the reflected and direct signals was set to $\alpha_{w}^{2}=0.7$. The satellite elevation measurements correspond to the satellites in view at Calais, France (50° 55′ 14.093″ N, 1° 56′ 59.44″ E), on 25 September 2013. The root-mean-square error (RMSE) was computed for 120 observation periods of 10 min, each period starting one minute later than the previous one, with *N* = 600 observations taken at one sample/s. We assume here that we are in a classic bistatic configuration depicted in Figure 1, where the receiver antenna is located at *h* = 2 m above the ground. The parameter *ϕ_R_* is defined by Equation (2), and the height *h* is sought with a resolution step of 1 × 10^−3^ m in a bounded search space *h_space_* = [0,5] m.

**Figure 8.**
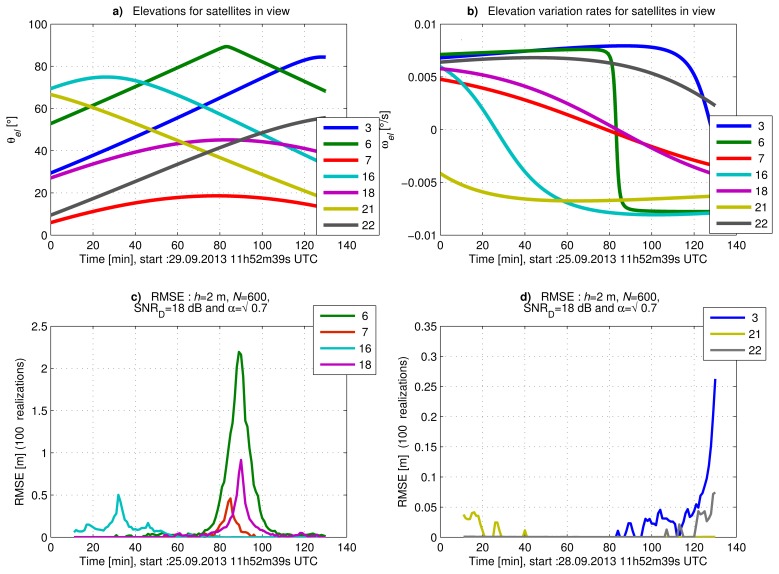
RMSE obtained for height estimation, with the corresponding elevation and elevation rate, for several satellites in view on September 25, 2013.

In Figure 8a,b, we show the satellites' elevations (*θ_el_*) and their elevation rates (*ω_el_*), respectively, as a function of time. We present in Figure 8c,d the RMSE of *ĥ* for the proposed estimator as a function of time, computed using the Monte Carlo method with 100 realizations. Figure 8c shows the RMSE of the satellites reaching an elevation rate close to zero at some instant during the full observation interval. Figure 8d shows the RMSE of the satellites with a high elevation rate, except for the end of the observation interval.

We observe in Figure 8c that the RMSE increases when the satellite elevation rate decreases to less than 2 × 10^−3^ °/s. In the case of Satellite 6, the RMSE is higher, because the satellite elevation is close to 90°. We observe in Figure 8d that the RMSE of Satellites 3 and 22 increases for high elevations. This is due to a low elevation rate and, so, a low frequency of the SNR evolution. These results are in accordance with the CRLB study in Section 4.1.

Let us now compute the RMSE of *ĥ* for different SNR values and observation interval durations. As before, the SNR refers to the signal-to-noise ratio for the direct signal, and the power ratio between the direct and reflected signals is kept at $\alpha_{w}^{2}=0.7$. In Tables 1, 2 and 3, we report the RMSE obtained with 1000 realizations of the noisy *A_G_* signal, for observation intervals of 600 s, 300 s and 150 s, respectively. The duration of these observation intervals was selected considering that for a satellite elevation of 35° and an elevation rate *ω_el_* = 6.8 × 10^−3^ °/s (e.g., Satellite 3 at 12h02 UTC, in Figure 8), a complete period of signal is observed after 600 s, a half a period after 300 s and a quarter of a period after 150 s.

From the results in Tables 1, 2 and 3, we observe that the proposed estimator is consistent and that the RMSE increases when the SNR decreases, which was expected. In these tables, we have defined two different sets of satellites. The first set includes Satellites 3, 6, 21 and 22 (in bold in the Tables) with a high absolute elevation rate *ω_el_* ≥ 6 × 10^−3^ °/s. Satellites 3, 21 and 22 have low initial elevations between 20° and 35°, and Satellite 6 has a high elevation, superior to 70°. In the second set, we consider the Satellites 7, 16 and 18, with a low *ω_el_*. Satellite 7 has a low initial elevation of 18°; Satellite 18 has an initial elevation of 44°; and Satellite 16 has a high initial elevation of 75°.

For Satellites 3, 21 and 22, the RMSE values presented in Tables 1 and 2 approach the CRLB values computed in Section 4.1. In these cases, we reach centimeter accuracy due to the high elevation rate of these satellites. However, for a period of observation of 300 s and a *C*/*N*_0_ = 35 dB-Hz, we reach just decimeter accuracy, showing the limits of this technique. In Table 3, we see that centimeter accuracy is not reached for an observation interval of 150 s, even with *C*/*N*_0_ = 50 dB-Hz. For 150 s, the SNR evolution covers only a short part of the oscillation period during the interval of observation and can be considered monotonic. In this context, we observe in Table 3 that the RMSE strongly depends on the considered part of the SNR cosine evolution rather than on the satellite's *ω_el_* (e.g., Satellites 3, 6, 21 and 22).

For Satellite 6, the results shown in Tables 1 and 2 are less accurate despite the fact that its *ω_el_* is similar to those of Satellites 3, 21 and 22. This can be explained by the higher elevation of Satellite 6. This result is indeed in accordance with the CRLB study (Section 4.1), because in this case, the SNR evolves with a lower frequency, so we do not cover a full period of the SNR variation over the observation interval.

For Satellites 7, 16 and 18, the results shown in Tables 1, 2 and 3 are also in accordance with the expected accuracy from the CRLB study. In this case, we reach just meter accuracy, because we observe only a small fraction of the SNR variation period due to the the low *θ_el_* of the satellites.

## 5. Experimental Framework

### 5.1. Experimental Results

**Figure 9.**
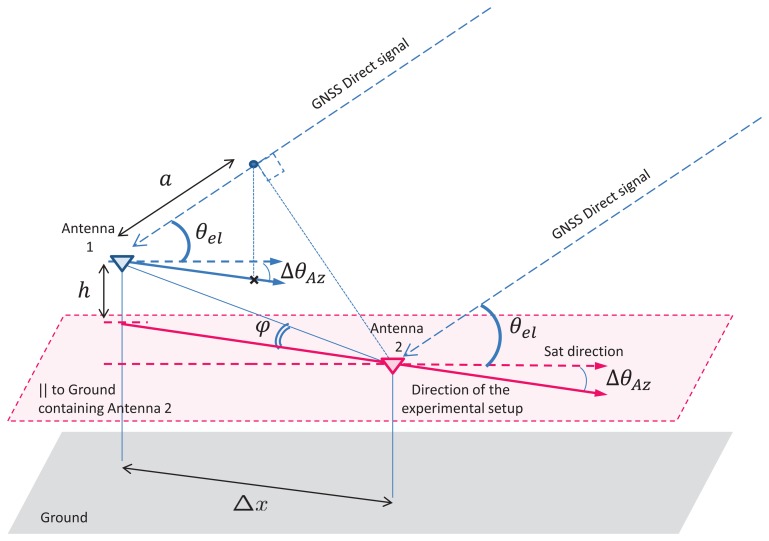
Geometry of the experiment.

In order to assess the proposed method, we constructed the following experimental setup to measure the height difference between two antennas, as depicted in Figure 9. The height difference is estimated with the interferometric approach described in Section 3. The advantage of the proposed assessment method is that we can have centimeter knowledge of the system geometry. Next, we derive the link between the height difference of the two antennas and the frequency of variation of the GNSS signal power.

For a height difference *h* between the two antennas, we have (see Appendix 2):

(23) $$a=\Delta x\cos\left(\theta_{el}\right)\cos\left(\Delta \theta_{Az}\right)-h\sin\left(\theta_{el}\right),$$

(24) h=Δxtan(φ),

where *a* is the path difference between the GNSS signals for the two antennas, Δ*x* the distance between the two antennas in the ground plane, *θ_el_* the elevation for the satellite in view and *ϕ* the angle between the two antennas and the ground plane. The paraxial approximation is assumed, so that the satellite signal arrives at both antennas with the same elevation angle, *θ_el_*. Δ*θ_Az_* is the angle between the vertical plane containing the two antennas and the vertical plane containing the satellite and any of the antennas. Then, it follows that:

(25) $$\frac{a}{h}=\frac{\cos\theta_{el}\cos\Delta \theta_{Az}}{\tan\varphi }-\sin\theta_{el}$$

(26) $$=\sqrt{\frac{\cos^{2}\Delta \theta_{Az}}{\tan^{2}\varphi }+1}\sin(\theta_{el}+\arctan*\left(\frac{\cos\Delta \theta_{Az}}{\tan\varphi }-1\right))$$

(27) $$=K\sin\left(\theta_{el}+K_{0}\right)$$

with:

(28) $$K=\sqrt{\frac{\cos^{2}\Delta \theta_{Az}}{\tan^{2}\varphi }+1,}$$

(29) $$K_{0}=\arctan*\left(\frac{\cos\Delta \theta_{Az}}{\tan\varphi }-1\right)$$

where arctan* () is the quadrant-specific inverse of the tangent. Finally, we can write:

(30) $$a=h\;K\;\sin\left(\theta_{el}+K_{0}\right)$$

The phase delay between the direct signal received by the two antennas, $\phi_{R}^{\exp}$, can be expressed as:

(31) $$\phi_{R}^{\text{exp}}=\frac{2\pi }{\lambda }a=\frac{2\pi }{c}f_{L1}a=\frac{2\pi }{c}f_{L1}h\;\left(\frac{\cos\theta_{el}\;\cos\Delta \theta_{Az}}{\tan\varphi }-\sin\theta_{el}\right)=\frac{2\pi }{c}f_{L1}h\;K\;\sin\left(\theta_{el}+K_{0}\right)$$

$\phi_{R}^{\exp}$ depends on *θ_el_* and on the satellite azimuth, with Δ*θ_Az_* assumed to be constant over the entire observation time. We then can conclude that $\phi_{R}^{\exp}$ evolves linearly, with a slope $\beta^{\exp}=\frac{2\pi }{c}f_{L1}h$, as a function of *K* sin(*θ_el_* + *K_0_*). Finally, $\beta^{\exp}=\frac{1}{2}\beta$, where *β* is the frequency of the variation of the SNR as a function of sin(*θ_el_*) in the reflection scenario described in Section 2.

### 5.2. Assessment with Real Data

**Figure 10.**
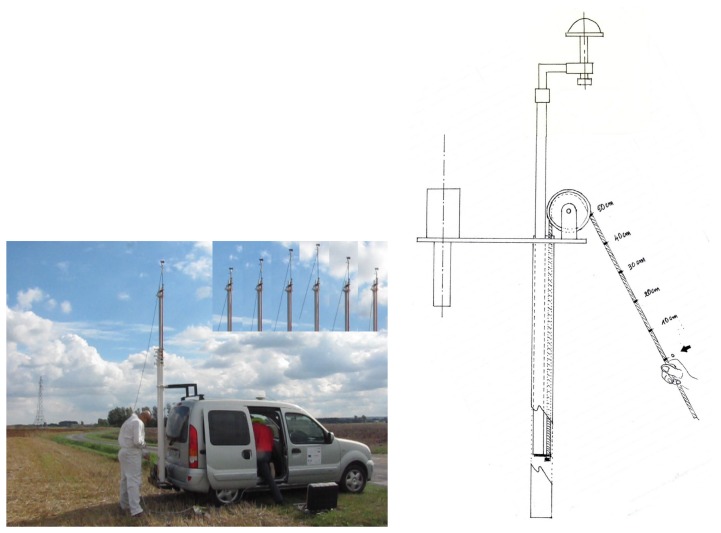
The vehicle and its telescopic mast.

Figure 10 shows the experimental vehicle and the telescopic mast used. The figure also shows the system that gives us the ability to precisely change the height of the antenna installed on the top of the mast. The second direct antenna is situated on the roof of the car at a horizontal distance of Δ*x* = 1.92 m. The height *h* between the two antennas is known, so we can derive the value of *ϕ* and define the complete geometry of the experimental system. We used the Novatel OEM4-G2 ProPak RT2W (GPS + WAAS/EGNOS) commercial receiver [21]. The two antennas were connected using a passive RF-combiner. The *C*/*N_0_* measurements, as well as the satellite elevation values were provided by the receiver at a rate of 1 Hz.

**Figure 11.**
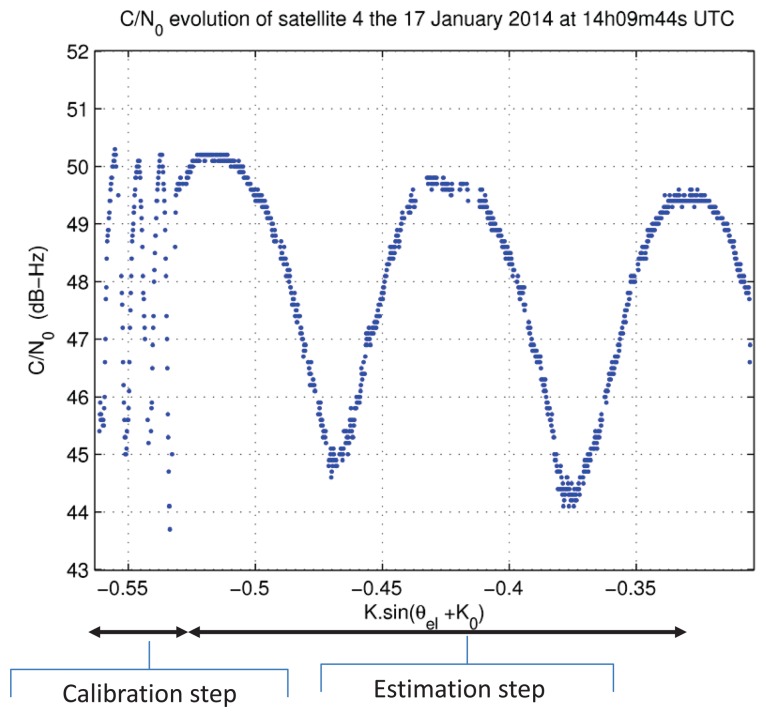
Evolution of *C/N_0_* for *h_ref_* = 2.13 m and Δ*x* = 1.92 m.

We show in Figure 11 an example of *C*/*N*_0_ evolution as a function of of *K* sin(*θ_el_* + *K*_0_). In this figure, we differentiate two periods of time in the signal. These two periods correspond to two different processing steps: the calibration step and the estimation step.

**Figure 12.**
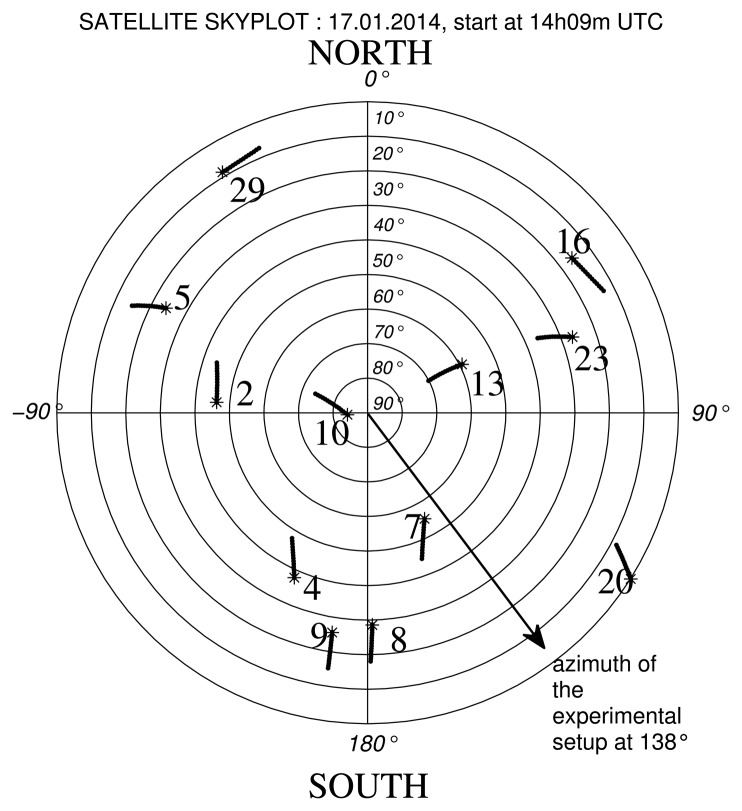
Trajectories of the satellites in view during the measurements, on 17 January 2014, at 14h09 UTC.

**Figure 13.**
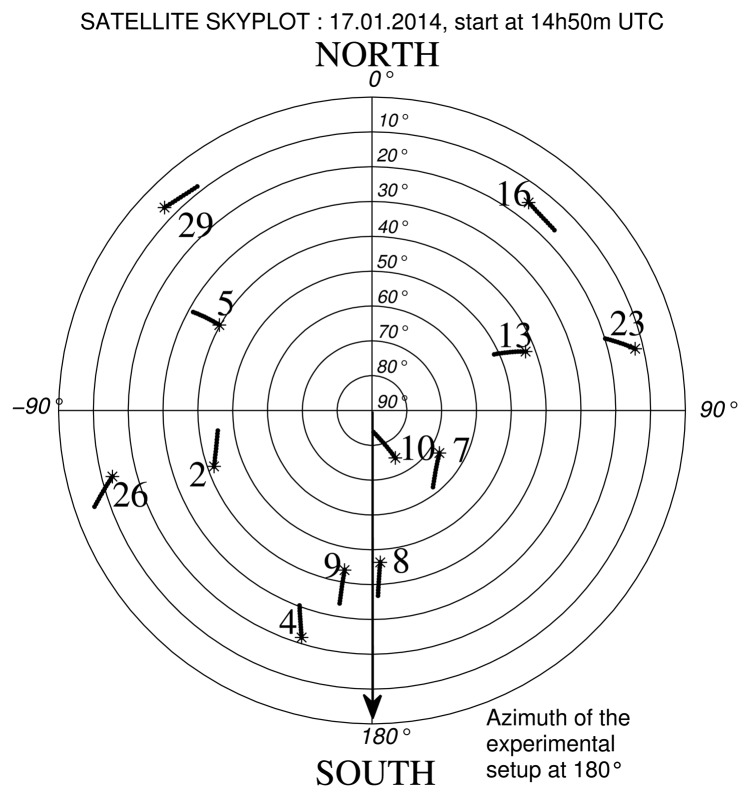
Trajectories of the satellites in view during the measurements on 17 January 2014, at 14h50 UTC.

We report in Tables 4 and 5 the estimated height (*ĥ*) obtained at Calais, France (50° 55′ 14.093″ N, 1° 56′ 59.44″ E), on 17 January 2014, with the proposed method. The reference height used was *h_ref_* = 2.13 m at 14h09 UTC and *h_ref_* = 8.24 m at 14h50 UTC. These heights were manually tape measured Figures 12 and 13 show the constellation of the visible satellites during the measurement periods and the direction of the experimental setup (working direction). We plotted in these figures the satellites' trajectories, with a star marking the end of each trajectory. In the experiments, *ĥ* was estimated with *N* = 600 observation samples and an search step resolution of 1 × 10^−3^ m in a bounded interval of *ĥ* = [*h_ref_* − 2, *h_ref_* + 2] m.

For *h_ref_* = 2 13 m the signals from Satellites 5 9 20 16 and 29 were not considered due to their low *C*/*N*_0_ and low elevation angles In these cases, the proposed estimator performed poorly, and the estimated height was far from *h_ref_*. The estimated height obtained for Satellites 4, 7, 10 and 23 showed a difference with the *h_ref_* between 2 cm and 9 cm. The mean estimated height is 2.15 m.

For *h_ref_* = 8.24 m, the signals from Satellites 16, 23, 26 and 29 were again not considered due to their low *C*/*N*_0_s. The results obtained with Satellites 2, 5, 8 and 9 are closer to *h_ref_*, with a difference between 1 cm and 5 cm. In this case, the mean estimated height is 8.25 m.

We can conclude that, after the calibration step, the proposed estimator can achieve centimeter accuracy under the experimental setup for a period of observation of 600 s.

## 6. Conclusions

In this article, we used an IPT to estimate the height between an antenna and a ground surface, where a GNSS signal has been reflected. We proposed to normalize the SNR measurements in order to construct a model of its evolution over time. The proposed estimator is based on two steps: A calibration step and an estimation step. The aim of the calibration step is to measure the maximum and minimum values of the SNR (or, equivalently, the *C*/*N*_0_) amplitude, in order to model the SNR variations for a bounded interval of possible surface heights.

The maximum likelihood estimate of the antenna height constructed with this nonlinear model was assessed in a study of the CRLB of the model. In this study, we showed that the accuracy of the estimation can be defined as a function of the satellite elevation, the elevation rate, the *C*/*N*_0_ and the power ratio between the direct and reflected signal.

In order to assess the method, we used synthetic data and verified that the proposed estimator is consistent with the number of observation samples and the *C*/*N*_0_ of the GNSS signal. We also showed that, using the proposed calibration step, we can expect centimeter accuracy with half a period of the SNR oscillation when we are in the best scenario. These conditions were identified using the CRLB.

Finally, we proposed an experimental framework that uses two direct signals received in different locations. The results using real data from field measurements showed that the proposed estimator can provide centimeter accuracy for a period of observation of 10 minutes within this framework.

## Figures and Tables

**Table 1.** RMSE of the estimated height *ĥ*, in meters, for an observation interval of 600 s. Reference height *h* = 2 m and $\alpha_{w}=\sqrt{0.7}$

*SNR*	18 dB	13 dB	8 dB
Satellite			
**Sat 3 at 11h57UTC**	0.001	0.001	0.027
**Sat 6 at 12h32 UTC**	0.005	0.016	0.090
**Sat 21 at 13h52 UTC**	0.001	0.001	0.014
**Sat 22 at 12h42 UTC**	0.001	0.001	0.045
Sat 7 at 13h22 UTC	0.072	0.131	0.432
Sat 16 at 12h12 UTC	0.107	0.143	0.596
Sat 18 at 13h12 UTC	0.098	0.1302	0.484

**Table 2.** RMSE of the estimated height *ĥ*, in meters, for an observation interval of 300 s. Reference height *h* = 2 m and $\alpha_{w}=\sqrt{0.7}.$

*SNR*	18 dB	13 dB	8 dB
Satellite			
**Sat 3 at 11h57 UTC**	0.005	0.027	0.152
**Sat 6 at 12h32 UTC**	0.059	0.080	0.286
**Sat 21 at 13h52 UTC**	0.001	0.018	0.224
**Sat 22 at 12h42 UTC**	0.001	0.011	0.152
Sat 7 at 13h22 UTC	0.183	0.245	0.849
Sat 16 at 12h12 UTC	0.232	0.313	1.162
Sat 18 at 13h12 UTC	0.457	0.625	1.558

**Table 3.** RMSE of the estimated height *ĥ*, in meters, for an observation interval of 150 s. Reference height *h* = 2 m and $\alpha_{w}=\sqrt{0.7}.$

*SNR*	18 dB	13 dB	8 dB
Satellite			
**Sat 3 at 11h57 UTC**	0.116	0.153	0.681
**Sat 6 at 12h32 UTC**	0.146	0.198	0.708
**Sat 21 at 13h52 UTC**	0.067	0.111	0.431
**Sat 22 at 12h42 UTC**	0.123	0.168	0.644
Sat 7 at 13h22 UTC	0.412	0.553	1.473
Sat 16 at 12h12 UTC	0.685	0.937	1.889
Sat 18 at 13h12 UTC	1.340	1.570	2.464

**Table 4.** 17 January 2014, at 14h09 UTC. Reference height: *h* = 2.13 m, Δ*x* = 1.92 m.

Satellite PRN	Estimated height (*ĥ*) (m)	|*K* sin(*θ_el_* + *K*_0_)| mean variation (s^−1^)	*C*/*N*_0,_*_min_* (dB-Hz)	*C*/*N*_0,_*_max_* (dB-Hz)	Comment
2**	2.86	7.0 × 10^−5^	43.8	50.5	low variation of |*K* sin(*θ_el_* + *K*_0_)|
4	2.22	1.7 ×10^−4^	44.8	50.2	
7	2.16	1.5 × 10^−4^	45.6	49.8	
8*	1.93	1.6 × 10^−4^	39.5	47	low *C*/*N*_0_
10	2.10	1.0 × 10^−4^	47.5	51.4	
13**	1.53	9.6 × 10^−5^	48.4	51.2
16	2.11	1.4 × 10^−4^	35.5	45.3	low *C*/*N_0_*
23	2.19	1.5 × 10^−4^	44.1	49.8	

**Table 5.** 17 January 2014, at 14h09 UTC. Reference height: *h* = 8.24 m, Δ*x* = 1.92 m.

Satellite PRN	Estimated height (*ĥ*) (m)	|*K* sin(*θ_el_* + *K*_0_)| mean variation (s^−1^)	*C*/*N*_0,_*_min_* (dB-Hz)	*C*/*N*_0,_*_max_* (dB-Hz)	Comment
2	8.29	7.2 × 10^−5^	44.5	50.2	low variation of |*K* sin(*θ_el_* + *K*_0_)|
5	8.27	1 × 10^−4^	42.1	49.2	
7**	8.79	5.9 × 10^−5^	47.6	50.9	low variation of |*K* sin(*θ_el_* + *K*_0_)|
8	8.23	1.2 × 10^−4^	43.9	47.8	
9	8.19	1.3 × 10^−4^	44	49.1	
10**	8.92	5.5 × 10^−5^	48.4	51.2	low variation of |*K* sin(*θ_el_* + *K*_0_)|
13*	8.01	8.9 × 10^−5^	45.6	50.8	low variation of |*K* sin(*θ_el_* + *K*_0_)|

**Figure A1.**
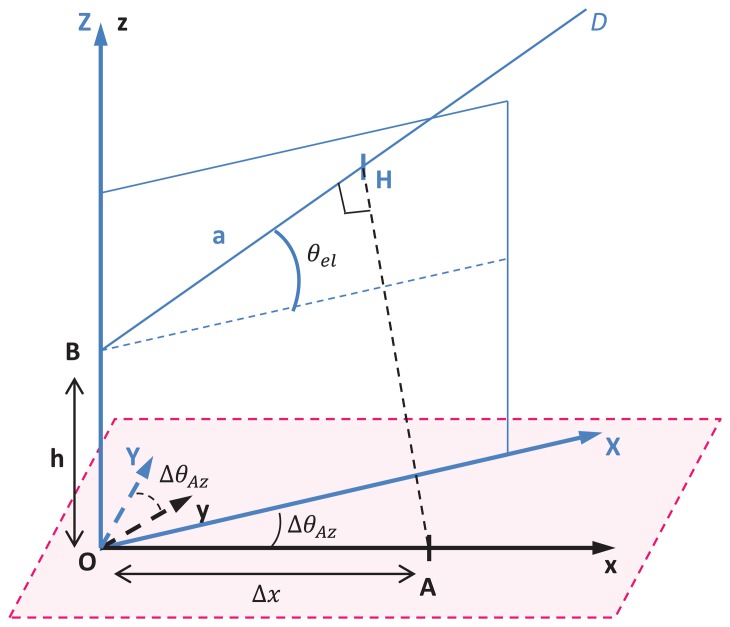
Geometry of the experimental framework.

## Appendix 1: FIM Computation

The Fisher information matrix (**I** (***ξ***)) for a deterministic parameter vector ***ξ*** is defined as [17]:

(32) $${\left[\text{I}(\text{ξ})\right]}_{ij}=-E\left[\frac{\partial^{2}\ln p\left(\text{x};\text{ξ}\right)}{\partial {\text{ξ}}_{i}\partial {\text{ξ}}_{j}}\right]$$

The observed samples are modeled as *y* [*n*; ***ξ***] = *A_G_* [*n*; ***θ***] + *w* [*n*], where *A_G_* [*n*; ***θ***] is the signal component and *w* [*n*] is zero-mean AWGN with variance $\sigma_{n}^{2}$. In general, for a vector signal parameter estimated in the presence of AWGN, Equation (32) yields:

(33) $${\left[\text{I}(\text{ξ})\right]}_{ij}=\frac{1}{\sigma_{n}^{2}}\sum_{n=0}^{N-1}\frac{\partial s\left[n;\text{ξ}\right]}{\partial {\text{ξ}}_{i}}\frac{\partial s\left[n;\text{ξ}\right]}{\partial {\text{ξ}}_{j}}$$

In Section 4.1, the signal model *A_G_* [*n*; ***ξ***] considered is:

(34) $$A_{G}\left[n;\theta \right]=A_{D}\sqrt{1+\alpha^{2}+2\alpha \cos\left(\gamma [n]h\right)}$$

where ***ξ*** = [*A_D_*, *α*, *h*]*^T^* is the unknown vector parameter.

Every element of the FIM, [**I**(***ξ***)]*_ij_*, is computed using a combination of the following partial derivatives of *A_G_*[*n*; ***θ***]:

(35) $$\begin{array}{l} \frac{\partial s\left[n;\text{ξ}\right]}{\partial A_{D}}=\frac{1}{A_{D}}A_{G}\left[n;\text{ξ}\right]\\ \frac{\partial s\left[n;\text{ξ}\right]}{\partial \alpha }=\frac{A_{D}^{2}\left(\alpha +\cos\left(\gamma [n]h\right)\right)}{A_{G}\left[n;\text{ξ}\right]}\\ \frac{\partial s\left[n;\text{ξ}\right]}{\partial h}=-\frac{A_{D}^{2}\alpha \gamma [n]\sin\left(\gamma [n]h\right)}{A_{G}\left[n;\text{ξ}\right]} \end{array}$$

In this way, the FIM is defined as a symmetric 3 × 3 matrix as follows:

(36) $$\text{I}(\text{ξ})=\frac{1}{\sigma_{n}^{2}}\left[\begin{array}{ccc} \frac{1}{A_{D}^{2}}\overset{N-1}{\underset{n=0}{\text{∑}}}A_{G}^{2}\left[n;\text{ξ}\right] & A_{D}\overset{N-1}{\underset{n=0}{\text{∑}}}\left(\alpha +\cos\left(\gamma [n]h\right)\right) & -A_{D}\alpha \overset{N-1}{\underset{n=0}{\text{∑}}}\gamma [n]\sin\left(\gamma [n]h\right)\\ A_{D}\overset{N-1}{\underset{n=0}{\text{∑}}}\left(\alpha +\cos\left(\gamma [n]h\right)\right) & A_{D}^{4}\overset{N-1}{\underset{n=0}{\text{∑}}}{\left[\frac{\left(\alpha +\cos\left(\gamma [n]h\right)\right)}{A_{G}\left[n;\text{ξ}\right]}\right]}^{2} & -A_{D}^{4}\alpha \overset{N-1}{\underset{n=0}{\text{∑}}}\frac{\gamma [n]\left(\alpha +\cos\left(\gamma [n]h\right)\right)\sin\left(\gamma [n]h\right)}{A_{G}^{2}\left[n;\text{ξ}\right]}\\ -A_{D}\alpha \overset{N-1}{\underset{n=0}{\text{∑}}}\gamma [n]\sin\left(\gamma [n]h\right) & -A_{D}^{4}\alpha \overset{N-1}{\underset{n=0}{\text{∑}}}\frac{\gamma [n]\left(\alpha +\cos\left(\gamma [n]h\right)\right)\sin\left(\gamma [n]h\right)}{A_{G}^{2}\left[n;\text{ξ}\right]} & A_{D}^{4}\alpha^{2}\overset{N-1}{\underset{n=0}{\text{∑}}}{\left[\frac{\gamma [n]\sin\left(\gamma [n]h\right)}{A_{G}\left[n;\text{ξ}\right]}\right]}^{2} \end{array}\right]$$

## Appendix 2: Experimental Framework Geometry Computation

Figure A1 shows the geometry of the experimental framework and defines two frames, (O,x,y,z) and (O,X,Y,Z), for the geometry computation. The plane (O,x,z) contains the car antenna *A* and the mast antenna *B*. The plane (O,X,Z) contains the mast antenna and the satellite in view.

The relation between both frames is:

(37) $$\{\begin{array}{l} X=x\;\cos\Delta \theta_{Az}+y\;\sin\Delta \theta_{Az}\\ Y=-x\;\sin\Delta \theta_{Az}+y\;\cos\Delta \theta_{Az}\\ Z=z \end{array}$$

The path difference *a* is defined with point *H*, which corresponds to the orthogonal projection of point *A* over *BD*, where *BD* is the line from the mast antenna to the satellite in view We thus have *Y_H_* = 0, so *y_H_* = *x_H_* tan *θ_el_* and $X_{H}=\frac{x_{H}}{\cos\Delta \theta_{Az}}$.

$\frac{Z_{H}-Z_{B}}{X_{H}-X_{B}}=\tan\theta_{el}$, with *X_B_* = 0 and *Z_B_* = *h*, implies that *Z_H_* − *h* = *X_H_* tan *θ_el_* and $Z_{H}=z_{H}=\frac{x_{H}\tan\theta_{el}}{\cos\Delta \theta_{Az}}+h$.

(AH) and (BH) being orthogonal, $\vec{AH}\cdot \vec{BH}=0$, so:

(38) $$\left(x_{H}-\Delta x\right)x_{H}+y_{H}^{2}+z_{H}\left(z_{H}-h\right)=0$$

(39) $$\left(1+\tan^{2}\Delta \theta_{Az}+\frac{\tan^{2}\theta_{el}}{\cos^{2}\Delta \theta_{Az}}\right)\;x_{H}^{2}+\left(\frac{h\tan\theta_{el}}{\cos\Delta \theta_{Az}}-\Delta x\right)\;x_{H}=0$$

(40) $$\frac{1}{\cos^{2}\theta_{el}\;\cos^{2}\Delta \theta_{Az}}x_{H}=\Delta x-\frac{h\;\tan\theta_{el}}{\cos\Delta \theta_{Az}}$$

(41) $$x_{H}=\Delta x\;\cos^{2}\theta_{el}\;\cos^{2}\Delta \theta_{Az}-h\;\sin\theta_{el}\;\cos\theta_{el}\;\cos\Delta \theta_{Az}$$

(42) $$x_{H}=\left(\Delta x\;\cos\theta_{el}\;\cos\Delta \theta_{Az}-h\;\sin\theta_{el}\right)\;\cos\theta_{el}\;\cos\Delta \theta_{Az}.$$

Finally,

(43) $${\left\|BH\right\|}^{2}=x_{H}^{2}+y_{H}^{2}+{\left(z_{H}-h\right)}^{2}$$

(44) $$=\frac{x_{H}^{2}}{\cos^{2}\theta_{el}\;\cos^{2}\Delta \theta_{Az}}$$

(45) $$={\left(\Delta x\;\cos\theta_{el}\;\cos\Delta \theta_{Az}-h\;\sin\theta_{el}\right)}^{2}$$

And:

(46) $$a=\left\|BH\right\|=\left|\Delta x\;\cos\theta_{el}\;\cos\Delta \theta_{Az}-h\;\sin\theta_{el}\right|$$
